# High-Sensitivity, Low-Hysteresis, Flexible Humidity Sensors Based on Carboxyl-Functionalized Reduced-Graphene Oxide/Ag Nanoclusters

**DOI:** 10.3390/nano15110800

**Published:** 2025-05-27

**Authors:** Hongping Liang, Lanpeng Guo, Yue Niu, Zilun Tang, Zhenting Zhao, Haijuan Mei, Ru Fang, Chen Liu, Weiping Gong

**Affiliations:** 1 Guangdong Provincial Key Laboratory of Electronic Functional Materials and Devices, Huizhou University, Huizhou 516007, China; 2 School of Integrated Circuits, Wuhan National Laboratory for Optoelectronics, Optics Valley Laboratory, Huazhong University of Science and Technology, Wuhan 430074, China; 3 School of Physical Sciences, Great Bay University, Dongguan 523000, China; 4 School of Chemistry and Materials Engineering, Huizhou University, Huizhou 516007, China

**Keywords:** sensor, humidity, carboxyl-functionalized, graphene, Ag nanoclusters

## Abstract

The measurement of humidity is of great significance for precision instruments, semiconductor integrated circuits, and element manufacturing factories. The oxygen-containing groups and noble metals in graphene-based sensing materials can significantly influence their humidity-sensing performance. Herein, 1,3,5-benzenetricarboxylic acid-functionalized reduced graphene oxide (H3BTC-rGO) loaded with Ag nanocluster nanocomposites (H3BTC-rGO/Ag) was synthesized via a facile one-step reduction method. The H3BTC-rGO/Ag-based sensor exhibited excellent humidity-sensing performance, including a higher sensitivity of 88.9% and a faster response/recovery time of 9 s/16 s towards 50% RH than those of other GO-, rGO-, and H3BTC-rGO-based sensors. The proposed humidity sensor was tested in the range of 0% to 100% RH and showed excellent sensitivity even at a low relative humidity of 0–10% or a high relative humidity of 90–100%. In addition, the H3BTC-rGO/Ag-based sensor had excellent selectivity, reliable repeatability, and good stability over 30 days under different relative humidities. Compared with H3BTC-rGO-200, the H3BTC-rGO/Ag-0.25-based sensor exhibited a low hysteresis of less than ±5% RH. The high performance was ascribed to the high density of the carboxyl groups and good conductivity of H3BTC-rGO, as well as the catalytic role of the Ag nanoclusters, resulting in high water adsorption rates. The potential applications of the H3BTC-rGO/Ag-based humidity sensor in human exhalation monitoring are also discussed. This work provides a reference for the application of graphene-based flexible sensors in monitoring very wet and dry environments.

## 1. Introduction

The measurement and control of humidity are of great significance for precision instruments, semiconductor integrated circuits, and element manufacturing factories [1]. Humidity sensors are widely used in meteorological forecasting and medical and food processing to ensure product quality and reliability [2]. In the manufacturing of lithium battery packs, even a small deviation in humidity can cause short circuits, resulting in thermal runaway of the battery [3]. In addition, when people breathe (inhale and exhale), the humidity of the breathed gases changes. These changes can reflect the body’s movement status and are one of the most important health indicators [4,5,6]. There is an urgent need to develop reliable humidity sensors as water is essential for human production and health. Humidity sensors face two major challenges: the balance between measurement range and accuracy, and the significant hysteresis that occurs and causes measurement inaccuracies.

The developed humidity sensors, categorized by the quantity of the electrical output, include resistive [4] and capacitive sensors [7], ones with different frequency types [8,9], etc. The resistive humidity sensor is one of the most widely researched humidity sensors because of its reusability, low cost, easy large-scale production, and miniaturization potential [10]. Various materials, such as ceramic [11,12], organic polymers [13], semiconductors [14,15], and electrolytes [16], have been investigated as humidity-sensing materials [17]. In recent years, graphene has been extensively studied and applied in resistive humidity sensors because of its large specific surface area, strong adsorption capabilities, and inherent flexible properties [17,18].

Graphene oxide (GO), with its numerous hydrophilic oxygen-containing groups, can effectively adsorb water molecules [18,19,20]. Thus, GO-based sensing materials are considered good candidates for fabricating humidity sensors [21]. However, it is still a challenge to fabricate humidity sensors with a large measurement range and high accuracy [22]. Furthermore, reduced graphene oxide possesses poor hydrophilicity during water absorption and poor reusability after water absorption, which limits its application [23]. Previous studies showed that organic molecules modified with reduced graphene oxide can improve the sensitivity, response speed, and reliability of their humidity-sensing performance by optimizing the relevant properties [8,24]. However, although many modification methods have been explored, the use of carboxyl-rich organic molecules to modify reduced graphene oxide through supramolecular assembly has not been reported. Supramolecular assembly between reduced graphene oxide and carboxyl-rich organic molecules promises to further improve measurement accuracy and response speed [25]. However, the hysteresis is still unsatisfactory.

Silver (Ag) can increase the number of active sites for the adsorption of water molecules because of its excellent catalytic properties and electrical conductivity [10,26]. This allows Ag-modified graphene to trap water molecules more efficiently and generate stronger electrical signals when water molecules are adsorbed, which is essential for the accurate and rapid detection of water molecules. Various nanosilver structures, including Ag nanoparticles [26], Ag nanowires [27], and Ag nanoarrays [28], have been reported. For example, Wang et al. reported an ultrafast and highly stable humidity sensor based on naphthalene-1-sulfonic acid sodium salt-modified graphene and Ag nanoparticles [29]. Moreover, the one-pot facile co-reduction technique has been used to prepare reduced graphene oxide-supported Ag nanoparticle catalysts [30]. However, the assembly of Ag nanoclusters remains challenging. An electrochemical sensor for the ultrasensitive determination of a tumor biomarker was designed using a Ag nanocluster-sensing platform [31]. However, the effective preparation of Ag nanoclusters in a composite is a challenge for practical applications [32]. Thermal evaporation has been used as a controllable modification method to construct Ag nanocluster-decorated CuCrO_2_ hybridizations [33]. The uneven distribution of Ag nanoclusters can lead to inconsistent performance and an increase in the cost of the sensor [34]. Therefore, there is an urgent need to find a simple and efficient way to balance cost and performance.

In this study, we aimed to fabricate H3BTC-rGO/Ag nanocomposites via a facile one-step reduction method using 1,3,5-benzenetricarboxylic acid (H3BTC), silver nitrate, and graphene oxide as the substrates. This approach simplifies the fabrication process and is expected to endow the composite with carboxyl-rich and activation properties. We investigated the effect of the H3BTC content on the chemical structure of graphene. Additionally, the influence of adding Ag on the resistance change rate and other aspects of the humidity-sensing performance was explored. The results showed that the H3BTC-rGO/Ag-based sensor had an excellent sensing performance within a large measurement range (0–100% RH in the air) at room temperature (25 °C). The sensor exhibited a high sensitivity of 88.9% and a fast response/recovery time of 9 s/16 s for 50% RH. Further evidence is provided showing that the H3BTC-rGO/Ag-based humidity sensor can be used to monitor human respiration, such as normal, rapid, and deep breathing. It also has potential uses in human–machine interactions and artificial intelligence (AI) to support more intuitive and intelligent interactions.

## 2. Materials and Methods

### 2.1. Materials

Silver nitrate (AgNO_3_, 0.1 mol/L), hydrazine hydrate (N_2_H_4_·H_2_O, ≥80 wt%), sodium hydroxide (NaOH, 98%), and 1,3,5-benzenetricarboxylic acid (H3BTC, AR) were purchased from Alfa Aesar (Shanghai, China). NO_2_ and CO_2_ were purchased from Dalian Special Gases Co., Ltd. (Dalian, China). The ammonia (NH_3_, ≥25 wt% in water), ethanol (C_2_H_6_O, ≥99.7%), methanol (CH_4_O, ≥99.5%), and formaldehyde (CH_2_O, ≥37 wt% in water) were purchased from Tianjin Zhiyuan Chemical Reagent Co., Ltd. (Tianjin, China) and used as analytical grade reagents. All chemicals were used directly without further purification.

### 2.2. Preparation of Humidity Sensing Materials

#### 2.2.1. Preparation of H3BTC-Functionalized Reduced Graphene Oxide

Graphene oxide was synthesized from flake graphite by a modified Hummers’ method, as described in our previous works [35,36,37]. H3BTC-functionalized reduced graphene oxide (H3BTC-rGO) was synthesized by supramolecular chemical modification and chemical reduction of GO with 1,3,5-benzenetricarboxylic acid and hydrazine hydrate. Briefly, 100, 150, 200, and 250 mg H3BTC were dissolved in 40 mL deionized water under stirring. NaOH solution (10 wt%) was added to adjust the pH of the solution to 7. Then, 5 mL GO dispersion (1 mg/mL) was added to the mixed solution. After stirring for 1 h, 10 mL hydrazine hydrate solution (0.5 μL/mL in water) was added and stirred at 90 °C for another 1 h. The product was rinsed three times with deionized water by vacuum filtration to ensure that the free H3BTC, Na^+^, and hydrazine hydrate were completely removed. The obtained samples were re-dispersed in 10 mL deionized water and labeled as H3BTC-rGO-100, H3BTC-rGO-150, H3BTC-rGO-200, and H3BTC-rGO-250. Reduced graphene oxide (rGO) was prepared by the same method without the addition of H3BTC.

#### 2.2.2. Preparation of H3BTC-rGO/Ag Nanocomposites

In the preparation of H3BTC-rGO/Ag, 200 mg H3BTC was dissolved in 40 mL deionized water under stirring. A NaOH solution (10 wt%) was added dropwise to adjust the pH to 7. Then, 5 mL GO dispersion (1 mg/mL) and different concentrations of silver nitrate (0.05, 0.25, and 0.5 mmol) were added and stirred for 1 h. After the addition of 10 mL hydrazine hydrate solution (0.5 μL/mL in water), the mixture was stirred at 90 °C for another 1 h. Finally, the product was rinsed three times with deionized water by vacuum filtration to ensure that the free H3BTC, Na^+^, Ag^+^, NO_3_^−^, and hydrazine hydrate were completely removed. The obtained samples were re-dispersed in 10 mL deionized water and labeled as H3BTC-rGO/Ag-0.05, H3BTC-rGO/Ag-0.25, and H3BTC-rGO/Ag-0.5, respectively. The samples were lyophilized in a lyophilizer at −55 °C and 5 Pa for 48 h for subsequent experiments and characterization.

### 2.3. Fabrication and Measurements of the Humidity Sensors

The humidity sensors were prepared by drop-coating the sensing material on PET-based Au-interdigital electrodes (Au-IDEs). Specifically, 10 μL of the sensing material dispersion (1 mg/mL) was dropped on the surface of the Au-IDEs to obtain a uniform sensing layer (Figure S1). All sensing tests were performed by using the 8-channel test system. The real-time resistance changes in the humidity sensors were measured by a source measure unit (Keithley 2450) with an input voltage of 3.3 V. All sensing tests were carried out at room temperature (25 ± 3 °C). The relative humidity was produced by passing dry air at a flow rate of 500 ccm through the bubble chamber at room temperature (Figure S2). Varying relative humidity levels were attained by setting different ratios of air flowing through the bubble chamber. For high humidity settings, a heating sleeve was adopted to augment the amount of water molecules. Humidity was monitored and adjusted in real-time using high-precision commercial temperature and humidity sensors. The injection of volatile gases, including ammonia, ethanol, methanol, and formaldehyde, was obtained by volatilizing the corresponding compounds. In the respiratory monitoring experiment, the sensor was attached to a nebulization cup integrated with a breathing mask using adhesive tape and positioned 3 cm away from the volunteer’s mouth/nose (Figure S7). The experiment was conducted at a consistent temperature of 25 °C and a relative humidity of 40–50%. The sensitivity (*S*) is defined as the following Equation (1):

(1) $$\;S\;(\%)=/R_{0}-R_{t}\;/\;/\;R_{0}\times 100\%$$

where *R*_0_ is the resistance value in 0% RH and *R*_t_ is the resistance value in the fixed target relative humidity. The response/recovery time is defined as the time from humidity sensor contact with/away from the water molecules to the detection of 90% of the full-scale resistance change, respectively [38].

Relative humidity is defined as the ratio of the water contained in a given volume to the water required to saturate the same volume at a constant temperature and is expressed as Equation (2) [14]:

(2) $$\%\;\mathrm{RH}=\frac{Amount\;of\;water\;contained\;in\;a\;given\;volume}{Amount\;of\;water\;required\;to\;saturate\;the\;same\;volume}\times 100\%$$

The H_2_O concentration (C_H2O_) in ppm units under different relative humidity can be calculated according to below Equation (3):

(3) $$c_{H_{2}O}=\frac{p_{s}\times \mathrm{RH}}{p_{0}}\times 1000000$$

where *p*_s_ is the saturated water vapor pressure (2.339 kPa) and *p*_0_ is the average atmospheric pressure (101 kPa) at 20 °C.

### 2.4. Characterization

The structural properties of the as-prepared materials were characterized by Fourier transform infrared spectra (FTIR, Vertex 70, Bruker, Ettlingen, Germany) and a Raman spectrometer (Raman, inVia, Renishaw, Gloucestershire, UK) with an excitation wavelength of 633 nm. The crystalline structure and phase of the samples were measured by an X-ray diffractometer (XRD, SmartLab, Rigaku, Tokyo, Japan) with Cu Kα radiation (λ = 0.154 nm) in the range of 10–90°. The morphologies of the PET-based sensors were observed using a microscope (DVM6, Leica, Wetzlar, Germany). The morphologies of the H3BTC-rGO and H3BTC-rGO/Ag nanocomposites were observed by field emission scanning electron microscopy (FESEM, SU8010, Hitachi, Tokyo, Japan), transmission electron microscopy (TEM), and high-resolution transmission electron microscopy (HRTEM, JEM-F200, JEOL, Tokyo, Japan) equipped with energy dispersive spectroscopy (EDS).

## 3. Results and Discussion

**Figure 1:**
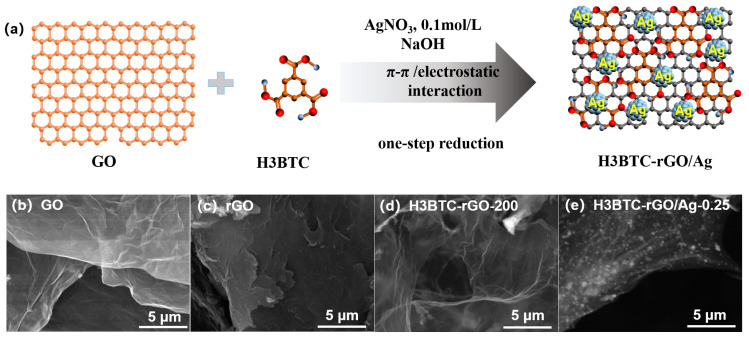
Schematic illustrations of (**a**) the sensing material preparation; SEM image of (**b**) GO, (**c**) rGO, (**d**) H3BTC-rGO-200, and (**e**) H3BTC-rGO/Ag-0.25 nanocomposites.

The schematic illustration for the preparation of the H3BTC-rGO/Ag nanocomposite is shown in Figure 1a. Graphene oxide and 1,3,5-benzenetricarboxylic acid (H3BTC) were mixed in deionized water under the assistance of NaOH, followed by the addition of a silver nitrate (AgNO_3_) solution. H3BTC was attached to the surface of GO by supramolecular assembly through π–π interactions. H3BTC with −COO^−^ provided anchor sites for Ag^+^ attachment through electrostatic interaction. Ag nanoclusters were grown on the H3BTC-rGO nanosheet surfaces by one-step reduction to obtain the H3BTC-rGO/Ag nanocomposites. The morphologies of the GO, rGO, H3BTC-rGO-200, and H3BTC-rGO/Ag-0.25 nanocomposites are displayed in Figure 1b-e. GO shows a sheet-like microstructure without a stacked structure, while rGO shows a seriously stacked structure (Figure 1b,c). After the supramolecular assembly and reduction of the graphene oxide, the obtained H3BTC-rGO nanosheets present a typical graphene wrinkle (Figure 1d). The morphology of the H3BTC-rGO/Ag-0.25 nanocomposites shows that the Ag nanoclusters are uniformly loaded on the H3BTC-rGO nanosheets (Figure 1e), which is favorable for increasing the number of adsorption and catalytic sites for water molecules and accelerating the electronic transmission from Ag to H3BTC-rGO.

### 3.1. Characterization of the Sensing Materials

H3BTC-rGO nanosheets with a large amount of negatively charged carboxylate radicals (−COO^−^) were successfully prepared by supramolecular assembly and reduction of graphene oxide using H3BTC and N_2_H_4_·H_2_O. As shown in Figure S3a–d, H3BTC-rGO-200 shows a large layered structure with typical graphene wrinkles, while H3BTC-rGO-100 and H3BTC-rGO-150 show a relatively intertwined structure with destruction. However, as the addition of H3BTC increases to 250 mg, the structure of H3BTC-rGO-250 has a more aggregated appearance. Different H3BTC-rGO/Ag nanocomposites were obtained at different concentrations of silver nitrate (0.05, 0.25, 0.5 mmol). The Ag nanoclusters are compactly loaded on the surfaces of the H3BTC-rGO nanosheets in the H3BTC-rGO/Ag nanocomposites (Figure S4a–c). Moreover, both the number and size of the Ag nanoclusters increase with the increase in the silver nitrate concentration. Typically, the Ag nanoclusters are uniformly distributed on the H3BTC-rGO matrix under 0.25 mmol silver nitrate (Figure S4b) compared to that below 0.05 mmol silver nitrate (Figure S4a). The loading of high-density Ag nanoclusters is attributed to the thin H3BTC-rGO-200 nanosheets and the anchoring sites provided by the −COO^−^ of H3BTC. However, when the silver nitrate concentration is increased to 0.5 mmol, the Ag nanoclusters become larger and show a higher degree of aggregation (Figure S4c).

**Figure 2:**
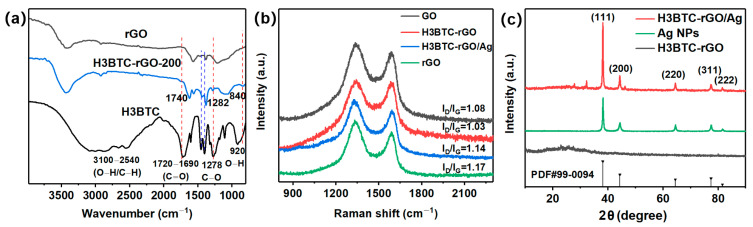
(**a**) FTIR spectra of rGO, H3BTC, and H3BTC-rGO-200. (**b**) Raman spectra of GO, H3BTC-rGO-200, H3BTC-rGO/Ag-0.25, and rGO. (**c**) XRD patterns for Ag, H3BTC-rGO-200, and H3BTC-rGO/Ag-0.25.

The FTIR spectra of rGO, H3BTC, and H3BTC-rGO-200 are shown in Figure 2a. The spectrum of H3BTC exhibits a strong peak at 1720–1690 cm^−1^, corresponding to the C=O stretching vibration in –COOH. The peaks at 1278 cm^−1^ and 920 cm^−1^ are assigned to the C–O stretching and O–H out-of-plane bending vibrations in –COOH. The broad peaks at 3100–2540 cm^−1^ are assigned to the –OH stretching vibration in –COOH and the C–H stretching vibration on the benzene ring. Compared with rGO, the characteristic peak of H3BTC appears in the spectrum of H3BTC-rGO-200, providing direct evidence for the successful assembly of H3BTC on rGO. As shown in Figure 2b, the Raman spectra of GO, H3BTC-rGO-200, H3BTC-rGO/Ag-0.25, and rGO show two major peaks at about 1336 cm^−1^ and 1586 cm^−1^, corresponding to the D-band and G-band of graphene, respectively [39]. The ratio of the intensities of the D- and G-bands (I_D_/I_G_) is related to the defect density. rGO and H3BTC-rGO/Ag-0.25 exhibit higher I_D_/I_G_ intensity than GO, indicating the successful reduction of GO and loading of Ag. It is interesting to note that H3BTC-rGO exhibits a lower intensity of the I_D_/I_G_ compared to GO and rGO, suggesting that H3BTC is able to repair the defect of rGO. The D-band positions of H3BTC-rGO/Ag-0.25 are redshifted by about 12 cm^−1^ compared to H3BTC-rGO, indicating that Ag was successfully loaded onto the H3BTC-rGO nanosheets through the electrostatic interaction [29]. The XRD results of H3BTC-rGO, Ag NPs, and H3BTC-rGO/Ag-0.25 are shown in Figure 2c. H3BTC-rGO shows a broad peak at 25°, indicating the successful reduction of GO [40,41,42]. The peaks at 38.1°, 44.3°, 64.4°, 72.3°, and 81.5° correspond to the (111), (200), (220), (311), and (222) planes of Ag, which are attributed to the crystalline phase of Ag in the H3BTC-rGO/Ag-0.25 [29].

**Figure 3:**
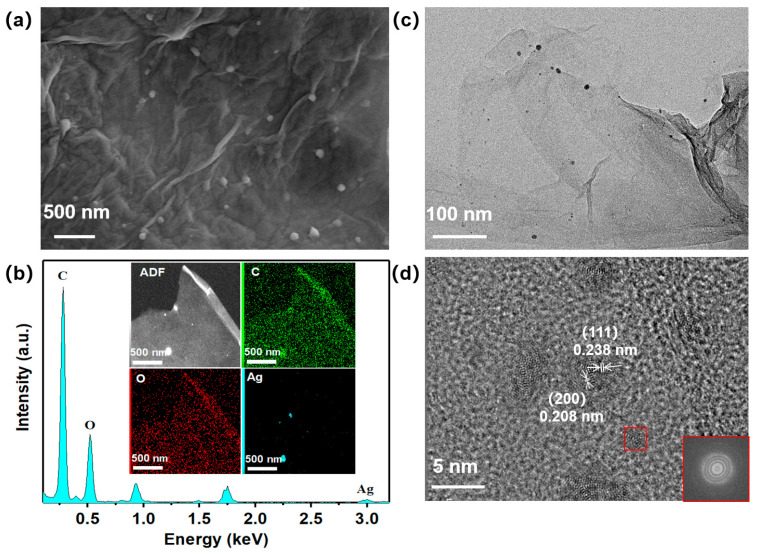
SEM images of (**a**) H3BTC-rGO/Ag-0.25 nanocomposites; EDS spectrum and C, O, Ag elemental mapping of (**b**) H3BTC-rGO/Ag-0.25 nanocomposites; TEM (**c**) and HRTEM (**d**) images of H3BTC-rGO/Ag-0.25 nanocomposites.

The uniformly dispersed Ag nanoclusters effectively improve the diffusion and interaction of water molecules on the sensing materials. The effective loading of Ag nanoclusters and their uniform distribution on the surface of the H3BTC-rGO nanosheets were characterized by various techniques. The surface morphology of H3BTC-rGO/Ag-0.25 is shown in Figure 3a. Note that the H3BTC-rGO nanosheets still retained their original morphologies after a one-step reduction. The EDS spectrum and corresponding elemental mapping of C, O, and Ag in H3BTC-rGO/Ag-0.25 confirmed the uniform decoration of Ag on the surface of the H3BTC-rGO nanosheets (Figure 3b). Figure 3c shows that the H3BTC-rGO/Ag-0.25 nanosheet structure is loaded with Ag nanoclusters and some larger Ag particles. The high-resolution TEM image shows that the diameter of the Ag nanoclusters is approximately 2–5 nm (Figure 3d).

### 3.2. Humidity Sensing Properties

**Figure 4:**
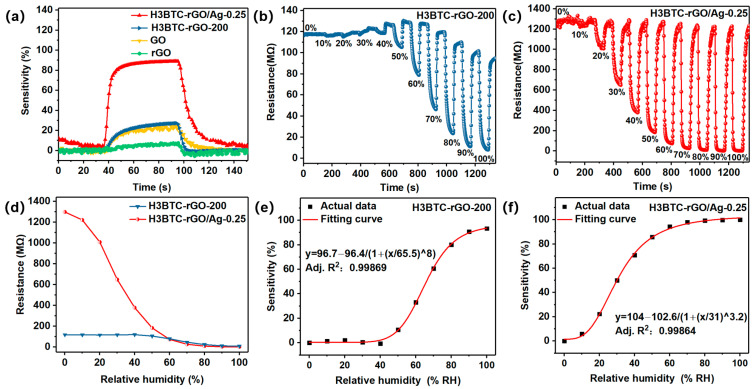
The sensitivity (**a**) of rGO, GO, H3BTC-rGO-200, and H3BTC-rGO/Ag-0.25 toward 50% RH. Successive response curves of the (**b**) H3BTC-rGO-200- and (**c**) H3BTC-rGO/Ag-0.25-based sensors exposed to 0~100% RH and the (**d**) corresponding resistance values of the H3BTC-rGO-200- and H3BTC-rGO/Ag-0.25-based sensors under 0~100% RH. The fitting curve of (**e**,**f**) sensitivity–relative humidity.

Figure S5a shows the dynamic response of H3BTC-RGO-100, H3BTC-RGO-150, H3BTC-RGO-200, and H3BTC-RGO-250 with the sensitivities of 21.5, 22.8, 27.3, and 24.3%, where the response/recovery times are 42/6, 41/5, 34/7, and 38/8 s, respectively (Table S1). H3BTC-RGO-200 has a higher response value and shorter response time compared to the other H3BTC-RGO composites. However, their response values and response times are still unsatisfactory. Therefore, H3BTC-RGO-200 was selected for the Ag nanocluster modification. The sensing characteristics of H3BTC-rGO/Ag-0.05, H3BTC-rGO/Ag-0.25, and H3BTC-rGO/Ag-0.5 for 50% RH are shown in Figure S5b. H3BTC-rGO/Ag-0.25 has the best comprehensive sensing performance, with a sensitivity of 88.9 and response/recovery times of 9/16 s (Table S1). As shown in Figure 4a, the sensitivity of H3BTC-rGO/Ag-0.25 was significantly higher than that of GO, rGO, and H3BTC-RGO-200. The sensitivity of H3BTC-rGO/Ag-0.25 toward 50% RH was 88.9%, whereas the sensitivities of GO, rGO, and H3BTC-RGO-200 were 24.1%, 7.6%, and 27.3%, respectively (Table S1). The improved humidity sensing performance can be ascribed to the good conductivity and the existence of the carboxyl group/Ag nanocluster in H3BTC-rGO/Ag-0.25. The successive response of H3BTC-rGO-200 and H3BTC-rGO/Ag-0.25 was measured under 0~100% RH during a continuous time, as shown in Figure 4b,c. The sensitivity of the H3BTC-rGO-200- and H3BTC-rGO/Ag-0.25-based sensors increases with the humidity increase. It is evident that within the range of 0~100% RH, H3BTC-rGO/Ag-0.25 exhibits a greater change in the resistance value compared to H3BTC-rGO-200 (Figure 4d). Moreover, the resistance of the H3BTC-rGO/Ag-based sensor reaches 330 kΩ at 100% RH, which is capable of detecting condensation. Figure 4e,f present the fitted plots illustrating the relationship between sensitivity and humidity. The fitting equations of the H3BTC-rGO-200- and H3BTC-rGO/Ag-0.25-based sensors are represented as y = 96.7 − 96.4/(1 + (x/65.5)^8^) and y = 104 − 102.6/(1 + (x/31)^3.2^), where y is sensitivity and x is relative humidity, with a correlation coefficient (R^2^) of 0.99869 and 0.99864, respectively.

**Figure 5:**
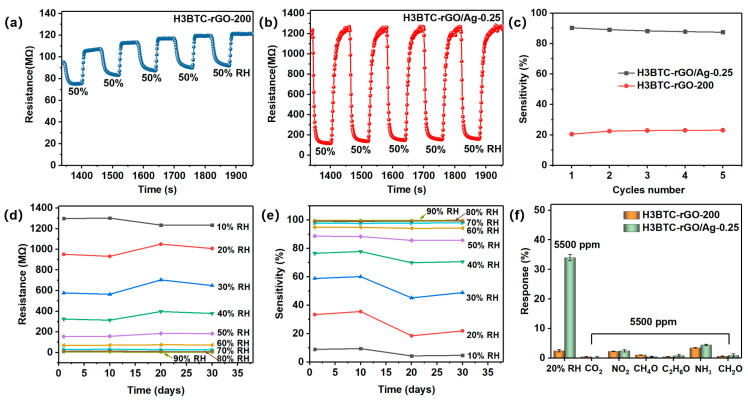
Repeated response of the (**a**) H3BTC-rGO-200- and (**b**) H3BTC-rGO/Ag-0.25-based sensors toward 50% RH and (**c**) their corresponding sensitivity within five cycles. Long-term stability of (**d**,**e**) the H3BTC-rGO/Ag-0.25-based sensor at 10%, 20%, 30%, 40%, 50%, 60%, 70%, 80%, and 90% RH. The selectivity (**f**) toward different interfering gases.

Five response−recovery cycles of the H3BTC-rGO-200- and H3BTC-rGO/Ag-0.25-based sensors toward 50% RH are shown in Figure 5a,b. As shown in Figure 5c, the sensitivity of each cycle is well maintained and agrees well with the fitting curves in Figure 4e,f. The response/recovery characteristic curves of the H3BTC-rGO/Ag-0.25-based sensor under 10% to 90% RH every 10 days were measured to evaluate the long-term stability (Figure S6). As shown in Figure 5d,e, the change rate of the resistance and sensitivity of H3BTC-rGO/Ag-0.25 shifted slightly, indicating that the sensors have good long-term stability. As shown in Figure 5f, H3BTC-rGO/Ag-0.25 shows strong anti-interference ability after exposure to 5500 ppm CO_2_, NO_2_, methanol, ethanol, NH_3_, and formaldehyde. Note that the sensitivity of H3BTC-rGO/Ag-0.25 toward 20% RH is 34%, while the sensitivities of the other interfering gases are all less than 4% at 5500 ppm, indicating the high selectivity of the H3BTC-rGO/Ag-0.25-based sensor toward humidity. As summarized in Table 1, the sensing performances of the flexible H3BTC-rGO/Ag-0.25-based sensor are competitive with some typical humidity sensors in terms of sensitivity, response/recovery time (9/16 s), and detection range (0–100% RH).

**Figure 6:**
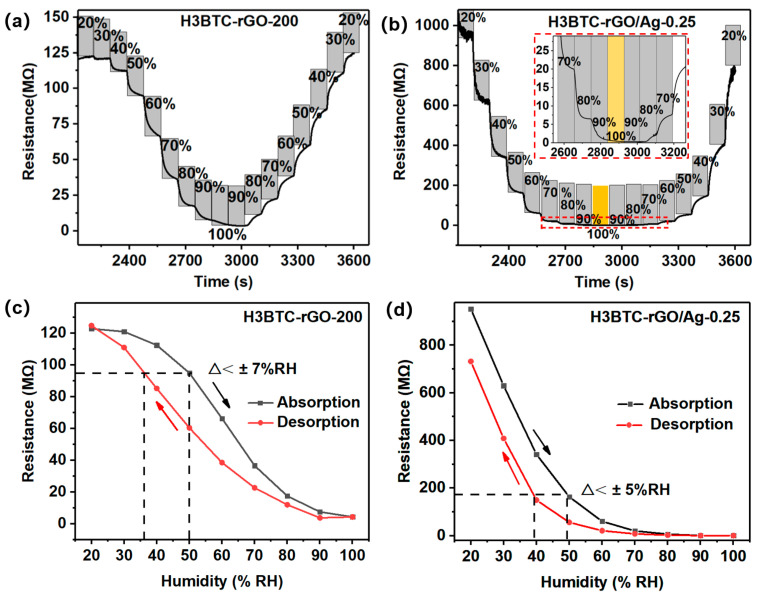
The resistance variation in the (**a**) H3BTC-rGO-200- and (**b**) H3BTC-rGO/Ag-0.25-based sensors under different relative humidity values (20%, 30%, 40%, 50%, 60%, 70%, 80%, 90%, and 100% RH). Hysteresis curve of the (**c**) H3BTC-rGO-200- and (**d**) H3BTC-rGO/Ag-0.25-based sensor.

Low hysteresis is an important factor for the high efficiency of humidity sensors [4]. The sensors were placed in a humidity chamber, and the humidity was increased from 20% RH to 100% RH and then decreased to 20% RH, with a step of 10% RH. The resistance variations in the H3BTC-rGO-200- and H3BTC-rGO/Ag-0.25-based sensors are shown in Figure 6a,b. The H3BTC-rGO/Ag-0.25-based sensor exhibits a hysteresis of less than ±5% RH (Figure 6d), while the H3BTC-rGO-200-based sensor shows a hysteresis of ±7% RH (Figure 6c). As shown in Figure 4c and Figure 6b, stable electrical signals can be observed in the sensing process over a wide humidity range, indicating that H3BTC-rGO/Ag-0.25 has great potential for application in full humidity sensors.

### 3.3. Application to Respiratory

**Figure 7:**
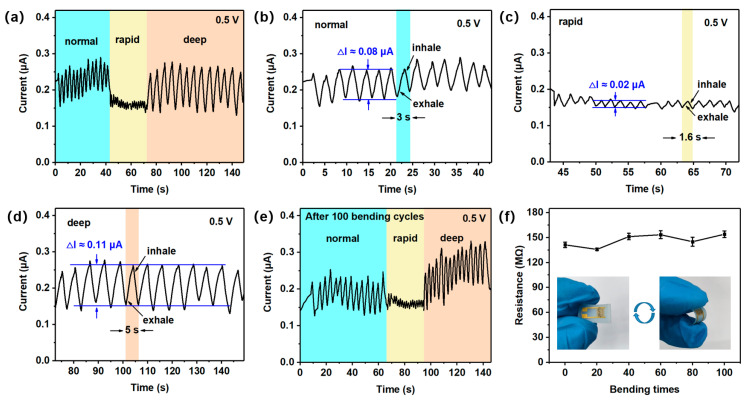
Response curves of (**a**) the human body under different breathing conditions. Response curves under (**b**) normal, (**c**) rapid, and (**d**) deep breathing conditions. Response curves of (**e**) the human body under different breathing conditions after 100 bending cycles. The resistance of (**f**) the H3BTC-rGO/Ag-based sensor at 55% RH after repeated bends.

With the development of wearable devices, new demands are being placed on flexible sensors for respiratory monitoring. The normal, rapid, and deep breathing rates of adults are approximately 12–20 times/min (3–5 s, one cycle), >20 times/min (<3 s, one cycle), and <12 times/min (>5 s, one cycle), respectively [47]. As shown in Figure 7a, simulated breathing tests were performed with three types of breathing (normal, rapid, and deep) using the detection device and measurement unit (Figure S7). The experimenter (a healthy 24-year-old female volunteer) first performed a normal breathing test and then performed a rapid breathing test after running 2 km, as well as imitated deep breathing by breathing slowly. The cycle of one rapid breath and one deep breath is approximately 1.6 s and 5 s, respectively, compared to that of normal breathing (3 s) (Figure 7b–d). There is also a clear difference in the breathing rate between normal, rapid, and deep breathing. Interestingly, the depth of breathing is at the same level (Figure 7a). Furthermore, the sensor maintained stable performance in the simulated breathing tests for normal, rapid, and deep breathing patterns after 100 bending cycles (Figure 7e). This confirms its reliability across diverse respiratory scenarios. Following 100 bending cycles, the sensor exhibited less than 9% resistance variation at 55% RH (Figure 7f), confirming its mechanical stability.

### 3.4. Humidity Sensing Mechanism

**Figure 8:**
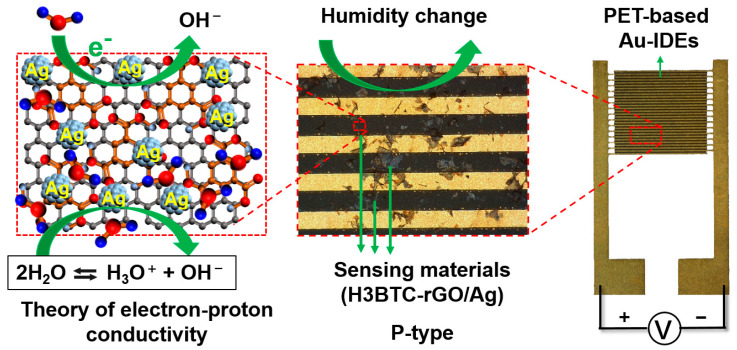
The sensing mechanism of H3BTC-rGO/Ag toward H_2_O molecules.

The humidity sensing mechanism of H3BTC-rGO/Ag is based on the change in electrical properties during the adsorption/desorption process. As shown in Figure 8, H3BTC-rGO/Ag, as a sensing material, was deposited onto PET-based Au-IDEs. H3BTC-rGO, with high conductivity, acted as electronic/charge transfer channels, while the Ag nanoclusters acted as the catalytic site. The introduction of carboxyl-rich organic molecules into graphene can reduce the conductivity of the material. At low humidity (RH < 10%), water molecules interact with H3BTC-rGO/Ag through diffusion. This hinders the movement of electrons and ions, resulting in a high resistance state (>1220.64 MΩ) of the sensor. As humidity increases, the adsorbed water molecules on the surface of the H3BTC-rGO/Ag reduce the resistance from 1220.64 MΩ to hundreds of MΩ through chemical adsorption. After reaching RH > 60%, the resistance value drops below 73.26 MΩ, and the water adsorption mechanism becomes primarily physical. The number of hydrated ions (H_3_O^+^) reaches the upper limit, and the reduction in resistance mainly depends on the change in the content of adsorbed water [48]. The carboxyl-rich organic molecules modified graphene with good conductivity, and the sufficient exposure of Ag nanocluster activity sites should be responsible for the enhanced humidity sensing performance in H3BTC-rGO/Ag.

## 4. Conclusions

In this work, carboxyl- and Ag-functionalized H3BTC-rGO/Ag nanocomposites were prepared by a facile one-step reduction method. FTIR, XRD, Raman, SEM, and TEM were used to investigate the structure and morphology characteristics of the H3BTC-rGO/Ag nanocomposites. A high-performance humidity sensor was fabricated using H3BTC-rGO/Ag as the sensing layer on the Au interdigital electrode of the flexible PET substrate. The obtained H3BTC-rGO/Ag-based humidity sensor exhibits excellent sensing performances in the range of 0–100% RH. The sensing results show that the resistance of the H3BTC-rGO/Ag-based sensor reaches 330 kΩ at 100% RH, which is capable of detecting condensation. In addition, the sensor exhibits high sensitivity (88.9%) with quick and full response/recovery (9 s/16 s) to 50% RH, as well as excellent selectivity, reliable repeatability, and long-term stability over 30 days. The improved humidity sensing performance of H3BTC-rGO/Ag is due to the synergistic effect of the hydroxyl groups and the Ag modification effect. H3BTC is used to harness the advantages of reduced oxide graphene, while the Ag nanoclusters are used to enhance the adsorption and catalytic behavior. Further evidence suggests that the H3BTC-rGO/Ag-based humidity sensor has potential applications in the healthcare industry for monitoring human respiration, including rapid, normal, and deep breathing. The proposed facile and low-cost fabrication strategy makes it a promising candidate for human breath detection.

## Figures and Tables

**Table 1:** Comparison of the humidity sensing performances for typical humidity sensors.

Materials	Sensor Type	S	T_res_ (s)/ T_rec_ (s)	Flexible	D_R_ (RH)
Ti_3_C_2_T_x_/K_2_Ti_4_O_9_ [43]	Resistance	1.49	65.2/84.8	no	11–95%
rGO-Ag scroll [10]	Resistance	908–1243	50/13	no	11–97%
CoFe_2_O_4_ [44]	Resistance	~590	25/2.6	no	8–97%
BL-G [45]	Resistance	5%	few seconds	no	30–70%
GO/BC [4]	Resistance	94%	13/47	no	5–85%
PDDA/GO [42]	Resistance	20.66%	94/134	yes	11–97%
Ti_3_C_2_T_x_/PDA [46]	Resistance	~85%	0.4/0.5	yes	5–95%
**H3BTC-rGO/Ag** **(This work)**	Resistance	**88.9%**	**9/16**	**yes**	**0–100%**

Sensitivity (S), response time (T_res_), recovery time (T_rec_), detection range (D_R_).

## Data Availability

The data are contained within this article and the Supplementary Materials.

## Supplementary Materials

The following supporting information can be downloaded at https://www.mdpi.com/article/10.3390/nano15110800/s1: Figure S1: Microscope image of H3BTC-rGO/Ag samples on PET-based Au-interdigital electrode; Figure S2: Schematic diagram of the dynamic gas sensing system; Figure S3: SEM image of (a) H3BTC-rGO-100, (b) H3BTC-rGO-150, (c) H3BTC-rGO-200, and (d) H3BTC-rGO-250 composites; Figure S4: SEM image of (a) H3BTC-rGO/Ag-0.05, (b) H3BTC-rGO/Ag-0.25, and (c) H3BTC-rGO/Ag-0.5 nanocomposites; Figure S5: The sensitivity (a) of H3BTC-rGO-100, H3BTC-rGO-150, H3BTC-rGO-200, and H3BTC-rGO-250 toward 50% RH and the sensitivity (b) of H3BTC-rGO/Ag-0.05, H3BTC-rGO/Ag-0.25, and H3BTC-rGO/Ag-0.5 toward 50% RH; Table S1: Comparison of the humidity sensing characteristics for the obtained sensors toward 50% RH; Figure S6: Response/recovery characteristic curves of the H3BTC-rGO/Ag-0.25-based sensor in 10% to 90% RH within 30 days; Figure S7: Photograph of the (a) human breath detection device (with breathing mask and the H3BTC-rGO/Ag-0.25 based sensor attached to the nebulization cup) and the (b) source measurement unit (Keithley 2450).
