# Supplementary material for: High-Sensitivity, Low-Hysteresis, Flexible Humidity Sensors Based on Carboxyl-Functionalized Reduced-Graphene Oxide/Ag Nanoclusters
Source: Nanomaterials (Basel). 2025 May 27;15(11):800. doi: 10.3390/nano15110800 (PMC12157789; doi:10.3390/nano15110800)
Supplement: Supplementary file 1 [file nanomaterials-15-00800-s001.zip › nanomaterials-3627115-supplementary.pdf]

Supporting Information

High-Sensitivity, Low-Hysteresis, Flexible Humidity Sensors Based on Carboxyl-Functionalized Reduced-Graphene Oxide/Ag Nanoclusters

Hongping Liang [1], Lanpeng Guo [2], Yue Niu [3], Zilun Tang [1, 4, *], Zhenting Zhao [1], Haijuan Mei [1], Ru Fang [1], Chen Liu [1], Weiping Gong [1, *]

[1] *Guangdong Provincial Key Laboratory of Electronic Functional Materials and Devices, Huizhou University, Huizhou 516007, China; lianghp@hzu.edu.cn (H.L.); zhzhting@hzu.edu.cn (Z.Z.); haijuanmei@hzu.edu.cn (H.M.); 2307080211@stu.hzu.edu.cn (R.F.); 2307080228@stu.hzu.edu.cn (C.L.)*

[2] *School of Integrated Circuits, Wuhan National Laboratory for Optoelectronics, Optics Valley Laboratory, Huazhong University of Science and Technology , Wuhan 430074, China; guolanpeng@hust.edu.cn*

[3] *School of Physical Sciences, Great Bay University, Dongguan 523000, China; niuyue@gbu.edu.cn*

[4] *School of Chemistry and Materials Engineering, Huizhou University, Huizhou 516007, China*

** Correspondence: tangzl@hzu.edu.cn (Z.T.); gwp@hzu.edu.cn (W.G.)*

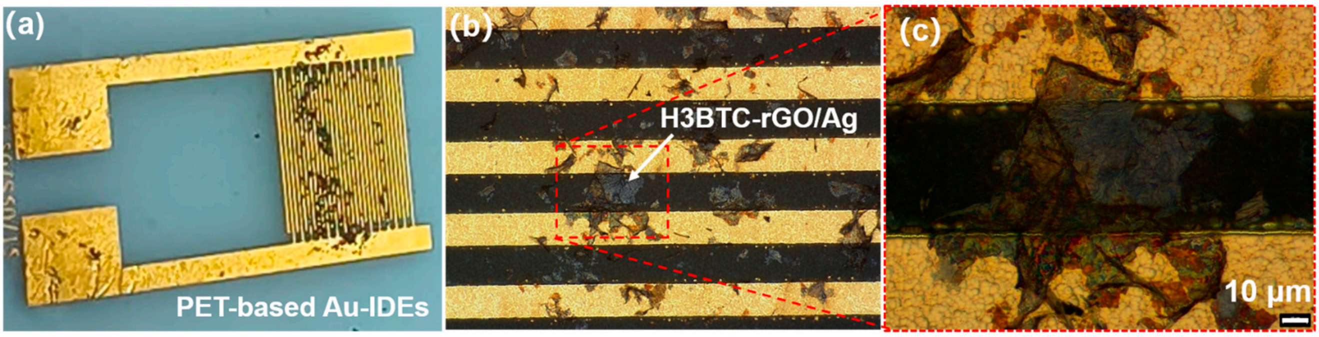

Figure S1. Microscope image of H3BTC-rGO/Ag samples on PET-based Au-interdigital electrode.

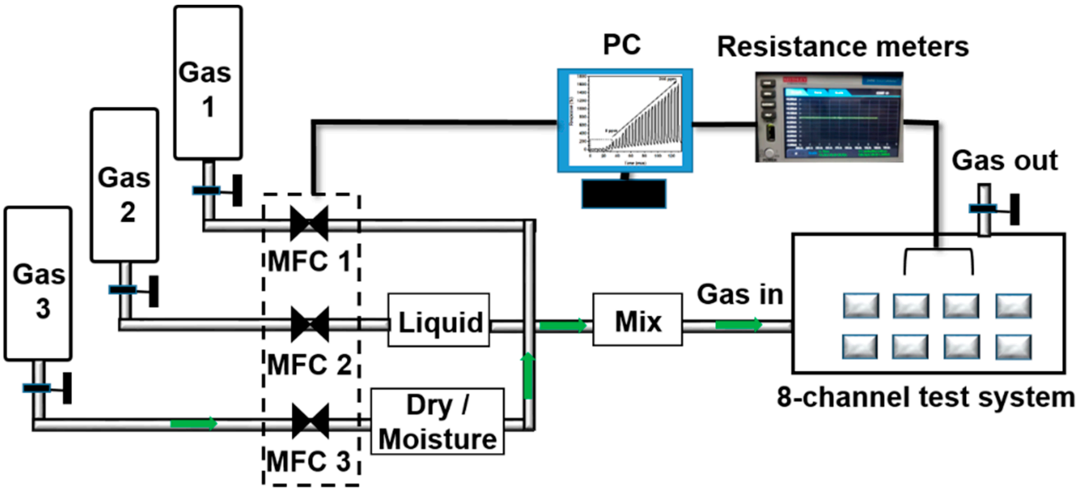

Figure S2. Schematic diagram of the dynamic gas sensing system.

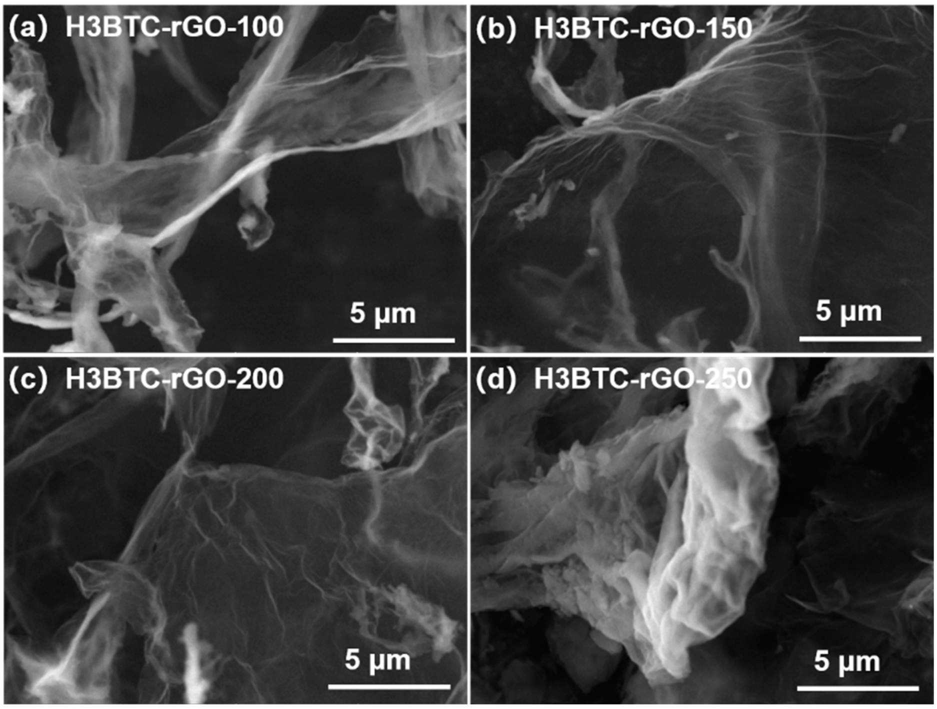

Figure S3. SEM image of (a) H3BTC-rGO-100, (b) H3BTC-rGO-150, (c) H3BTC-rGO-200, and (d) H3BTC-rGO-250 composites.

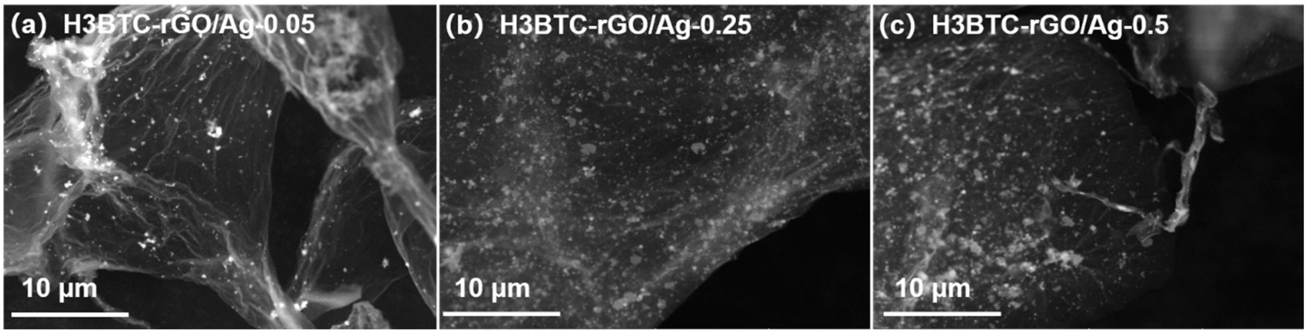

Figure S4. SEM image of (a) H3BTC-rGO/Ag-0.05, (b) H3BTC-rGO/Ag-0.25 and (c) H3BTC-rGO/Ag-0.5 nanocomposites.

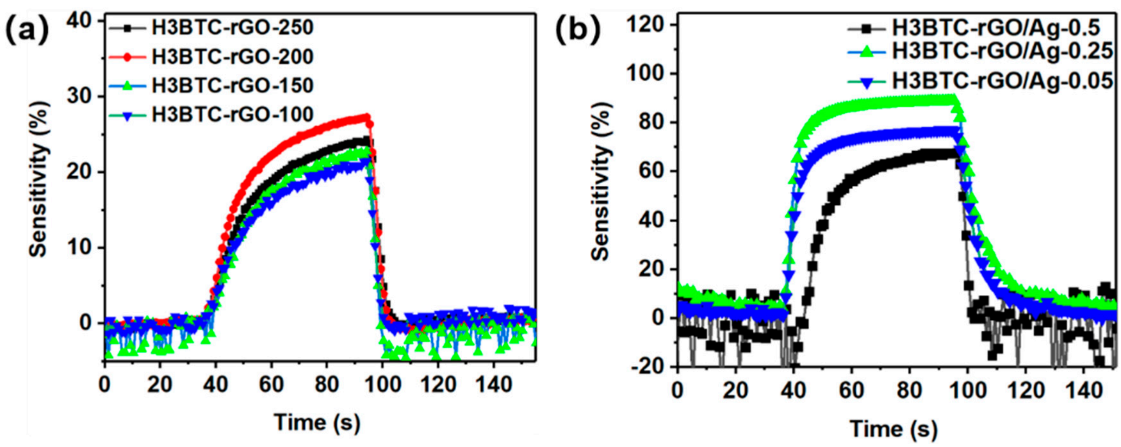

Figure S5. The sensitivity (a) of H3BTC-rGO-100, H3BTC-rGO-150, H3BTC-rGO-200, and H3BTC-rGO-250 toward 50% RH; The sensitivity (b) of H3BTC-rGO/Ag-0.05, H3BTC-rGO/Ag-0.25, and H3BTC-rGO/Ag-0.5 toward 50% RH.

Table S1 Comparison of the humidity sensing characteristics for the obtained sensors toward 50% RH

Materials	Sensitivity (%)	Response/recovery time (s)
**GO**	24.1	34/30
**rGO**	7.6	53/5
**H3BTC-rGO-100**	21.5	42/6
**H3BTC-rGO-150**	22.8	41/5
**H3BTC-rGO-200**	27.3	34/7
**H3BTC-rGO-250**	24.3	38/8
**H3BTC-rGO/Ag-0.05**	77.0	14/30
**H3BTC-rGO/Ag-0.25**	88.9	9/16
**H3BTC-rGO/Ag-0.5**	67.7	25/5

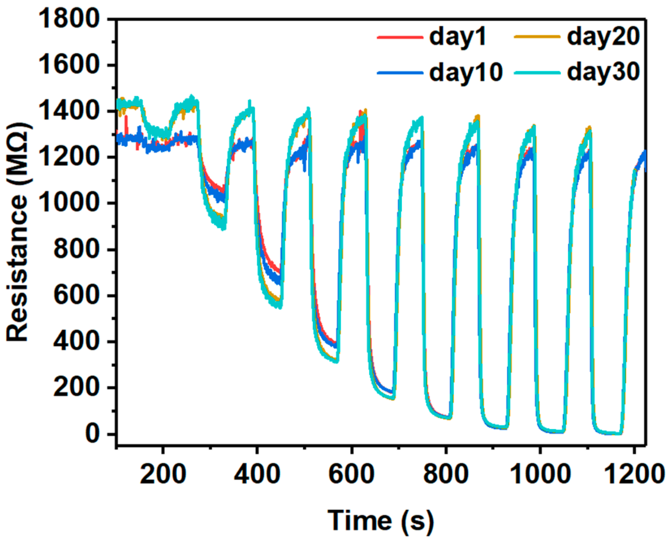

Figure S6. Response/recovery characteristic curves of H3BTC-rGO/Ag-0.25 based sensor in 10% to 90% RH within 30 days.

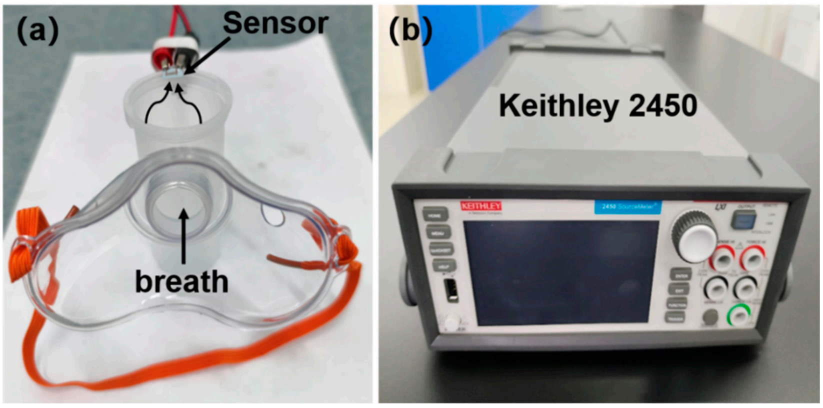

Figure S7. The photograph of the (a) human breath detection device (with breathing mask and the H3BTC-rGO/Ag-0.25 based sensor attached to the nebulization cup) and the (b) source measurement unit (Keithley 2450).
